# Examination of In Vivo Mutations in VP4 (VP8*) of the Rotarix^®^ Vaccine from Shedding of Children Living in the Amazon Region

**DOI:** 10.3390/v18010070

**Published:** 2026-01-03

**Authors:** Mauro França Silva, Beatriz Vieira da Silva, Emanuelle Ramalho, Yan Cardoso Pimenta, Leonardo Luiz Pimenta da Silva, Laricy da Silva Vieira, Maria da Penha Trindade Pinheiro Xavier, Alberto Ignacio Olivares Olivares, José Paulo Gagliardi Leite, Marcia Terezinha Baroni de Moraes

**Affiliations:** 1 Program in Tropical Medicine, Oswaldo Cruz Institute, Oswaldo Cruz Foundation, Fiocruz, Avenida Brasil, 4365-Manguinhos, Rio de Janeiro 21040-360, Brazil; mauro@bio.fiocruz.br (M.F.S.); vieirabeatriz029@gmail.com (B.V.d.S.); yancpimenta@gmail.com (Y.C.P.); 2 Technological Coordination, Tetraviral Vaccine, Immunobiological Technology Institute (Biomanguinhos), Oswaldo Cruz Foundation, Fiocruz, Avenida Brasil, 4365-Manguinhos, Rio de Janeiro 21040-360, Brazil; 3 Laboratory of Comparative and Environmental Virology, Oswaldo Cruz Institute, Oswaldo Cruz Foundation, Fiocruz, Avenida Brasil, 4365-Manguinhos, Rio de Janeiro 21040-360, Brazil; manu.bio.ramalho@gmail.com (E.R.); leonardolps974@gmail.com (L.L.P.d.S.); laricyvieira12@gmail.com (L.d.S.V.); dadade.xavier@gmail.com (M.d.P.T.P.X.); jpgleite@gmail.com (J.P.G.L.); 4 Secretaria Estadual de Saúde de Roraima, SESAU/RR, Rua Madrid, 180-Aeroporto, Boa Vista 69310-043, Brazil; albertouffr@gmail.com; 5 College of Medicine, State University of Roraima, Avenida Helio Campo, s/n-Centro, Caracaraí, Boa Vista 69360-000, Brazil

**Keywords:** acute gastroenteritis, Rotavirus A, Rotarix^®^, Histo Blood Group Antigen

## Abstract

Group A rotaviruses (RVAs) remain the leading cause of acute gastroenteritis (AGE) in young children in low- and middle-income countries. In Brazil, the oral attenuated RVA vaccine (Rotarix^®^), monovalent genotype G1P[8], is distributed by the national immunization program and has drastically reduced morbidity and mortality associated with RVA etiology. In this study, Rotarix^®^ G1P[8] was detected using specific qRT-PCR from the fecal shedding of children living in the Amazon region, and 18.3% (29/158) were positive and 75.8% (22/29) presented with AGE. The VP4 (VP8*) gene of these sheddings, submitted to Sanger nucleotide sequencing, showed an occurrence of mutations, including the silent mutation at 144C > G (one child) and the following missense mutations— 499T > C (F167L) (two children), 644G > C (C215S) (one child), and 787G > A (E263K) (one child). These mutations had no impact on the protein model structure in silico deduced from the VP4 (VP8*) mutants. The in silico protein model deduced from the VP4 (VP8*) nucleotide sequences, bound to type 1H sugar antigens (H1) and its precursor Lac-para-N-biose (LNB), had a stronger binding to the G1P[8] genotype, when compared to G3P[8]. Rotarix^®^ shedding was higher in HBGA secretors than in non-secretors (79.3%; 23/29). A total of 11.4% (18/158) of children with Rotarix^®^ G1P[8] shedding were unvaccinated, indicating the occurrence of indirect protection. Stability evidence of Rotarix^®^ VP4 (VP8*) spike protein from samples collected in vivo is presented.

## 1. Introduction

Rotavirus (RV) is the primary cause of severe gastroenteritis in children under five years of age and remains a significant global health concern [1,2]. Before the advent of RV vaccines, RV infections were a leading cause of severe pediatric acute gastroenteritis (AGE), contributing to approximately 500,000 deaths among children each year. They were implicated in 30% to 50% of all hospital admissions for AGE in children under the age of five, underscoring their substantial global burden prior to widespread immunization efforts [3]. In high-income countries with established vaccination programs, the incidence of RV infection has declined significantly. However, in low- and middle-income countries where vaccine coverage is limited, RV remains a leading cause of life-threatening diarrhea in young children [4]. Indeed, in Brazil, a country with a large territorial extension, RVA vaccine coverage (Rotarix^®^; Wavre, Belgium, GlaxoSmithKline) can be low in some regions, especially in the Amazon, with a coverage of 61–63% reported in recent years [5,6]. However, the success of vaccination in Brazil since its introduction in 2006 (the first country in South America) has resulted in a drastic reduction in morbidity and mortality from AGE in young children [7].

RV belongs to the *Sedoreoviridae* family, with a genome enclosed in a three-layer capsid, composed of 11 double-stranded RNA (dsRNA) segments that encode six structural proteins (VP1–VP4, VP6, and VP7) and six non-structural proteins (NSP1–NSP5/6) [8]. RVs are classified in a binary system based on the main neutralizing antigens, namely, the spike protein (VP4) and the outer capsid major glycoprotein (VP7) [9,10]. For the RV infection, the VP4 spike protein must be cleaved by a protease, which results in the proteins VP5* and VP8* [11]. Because they comprise the outer layer of the virion, VP4 and VP7 are capable of eliciting neutralizing antibodies [12]. Consequently, VP4 and VP7 are likely to be under strong selection for diversification to mediate cell entry or escape host immune responses [13,14,15]. The VP8* domain forms the distal globular head of the VP4 protein and plays a central role in the initial attachment of rotavirus to host cells. Structurally, VP8* adopts a conserved β-sandwich fold, while surface-exposed loops constitute the functional core involved in receptor recognition and genotype-specific interactions [16,17]. Structural and biochemical studies have demonstrated that the VP8* domain of human P[8] rotaviruses mediate binding to Histo-Blood Group Antigens (HBGAs), including H type 1 and lacto-N-biose (LNB), which act as attachment factors during viral entry [17,18]. Importantly, the HBGA-binding interface partially overlaps with antigenic regions recognized by neutralizing antibodies, indicating that amino acid variations within VP8* may influence both receptor binding and immune recognition [16,19]. Therefore, sequence polymorphisms observed in VP8*, even when structurally subtle, warrant structural evaluation to assess their potential impact on sugar interaction and viral fitness. Based on antibody reactivity and VP6 protein sequence identity, the recognized RV species include A, B, C, D, E, F, G, H, I, and J [2], among which species A is responsible for over 90% of infections, while species B and C are only sporadically detected in humans [20,21]. RVAs are classified into distinct genotypes—42 for VP7 (G types) and 58 for VP4 (P types) [21]. The globally dominant G/P-genotypes combinations, causing nearly 90% of human RVA infections, are G1P[8], G2P[4], G3P[8], G4P[8], G9P[8], and G12P[8] [22,23,24,25].

Several factors make RVAs a difficult pathogen to control, even with current vaccines. The propensity of rotavirus for co-infection and cross-pollination with other strains, which contributes to evolutionary dynamics, is one of the main factors. Furthermore, RVA genomes exhibit high mutation rates and undergo frequent rearrangements. Genomic rearrangements may also contribute to rotavirus diversity although they are not the primary factor in the evolution of these viruses, as recently reviewed [26].

Quantitative real-time reverse transcription PCR (qRT-PCR) has been employed for the detection of viral RNA in fecal samples for the identification of the etiological viral agent causing AGE [27]. Notably, qRT-PCR can show positive results of asymptomatic children under one year of age and is common in recently vaccinated children, particularly outside the hospital setting, who send fecal samples to laboratories without full clinical information. These RVA-positive children may be misinterpreted as vaccine failure (as wild-type virus) and potentially undermine public confidence in vaccine efficacy. Thus, diagnostic tests play a crucial role not only in clinical management but also in epidemiological surveillance and evaluation of vaccine performance. To accurately distinguish vaccine-derived strains from wild-type viruses, qRT-PCR to discriminate G1P[8] vaccine viruses from wild-type viruses could be useful. In Brazil, the Rotarix^®^ attenuated oral RVA vaccine, which is composed of a G1P[8] strain, is administered in a two-dose schedule, with dose 1 scheduled for 2 months of age (6–51 weeks) and dose 2 at 4 months of age (13–103 weeks).

HBGAs, present on the surface of the intestinal epithelium, function as receptors or adhesion factors and mediate susceptibility to RVA infection. The major determinant of HBGA-RVA susceptibility is a functional *FUT*2 gene that encodes the enzyme α (1,2)-fucosyltransferase, which uses fucose to create the H antigen. This process determines whether an individual is a “secretor” and will have this antigen and others (like A and B blood group antigens) in their saliva and other bodily fluids. “Non-secretors,” who lack the functional enzyme, do not secrete these antigens in their fluids, which can affect their susceptibility to RVAs [28]. Cellular attachment and entry, as well as HBGA binding in vitro, is mediated by the VP4 protein (P-genotype), which is post-translationally cleaved into a glycan binding domain and polypeptides [29]. As such, the P-genotypes determine the pattern of genetic susceptibility [29].

In this study, the qRT-PCR method of [30] was used to distinguish wild-type RVA G1P[8] samples from those derived from the vaccine (Rotarix^®^) with a cohort of children residing in the northwestern Amazon. Fecal samples were tested, and the vaccine (Rotarix^®^) was detected in previously unvaccinated children. The VP4 gene (VP8*) from these samples was submitted to nucleotide sequencing and protein in silico analysis, examining the impact of the detected mutations on the VP8* protein structure and interaction with the HBGAs.

## 2. Materials and Methods

### 2.1. Sampling

The samples in this study are part of a larger project to identify viral agents causing AGE and HBGA host genetic susceptibility, with approval from the Ethical Research Committee of the Federal University of Roraima (Approval number: CAAE 45542515.1.0000.5302, 23 November 2015). These are children under 5 years old who live in the northwestern Amazon region, in areas spanning Brazil, Venezuela, and Guyana, including demarcated indigenous territories in the Amazon rainforest. Fecal and saliva swab (containing mucosal cells) samples were collected in parallel in 2016 and 2017 from 734 children with (*n* = 485) and without AGE (controls) (*n* = 249). All these samples were tested for the presence of RVA (feces) and the definition of the secretory HBGA and Lewis (saliva) profiles, as previously described [5]. In 2021, 202 children (101 with and 101 without AGE) were also involved in this study; the detection of RVA and the HBGA profiles (secretory and Lewis) was verified in this study. The collection site of the samples was the emergency care unit at the “Hospital da Criança de Santo Antonio” (HCSA) located in Boa Vista, state of Roraima (RR, Brazil). The pediatrician participating in this study collected all samples immediately after the children’s admissions and examined them. Clinical inclusion criteria to AGE were according to the World Health Organization [31]: three liquid/semi-liquid evacuations in a 24 h period and dehydration. Some children were hospitalized due to the degree of malnutrition, with no deaths reported. The children’s parents or guardians were interviewed for data collection and completion of a form containing clinical and epidemiological information for each child. Immediately after collection, all samples were temporarily stored at −20 °C in the HCSA and then sent, together with clinical–epidemiological records, to the Laboratory of Comparative and Environmental Virology–Regional Rotavirus Reference Laboratory (LVCA-RRRL) under strict transport criteria to maintain the temperature and integrity of the samples. The LVCA-RRRL is part of the ongoing national network for AGE surveillance and is coordinated by the General Coordination of Public Health Laboratories of the Brazilian Ministry of Health.

### 2.2. Control Sampling

**Figure 1:**
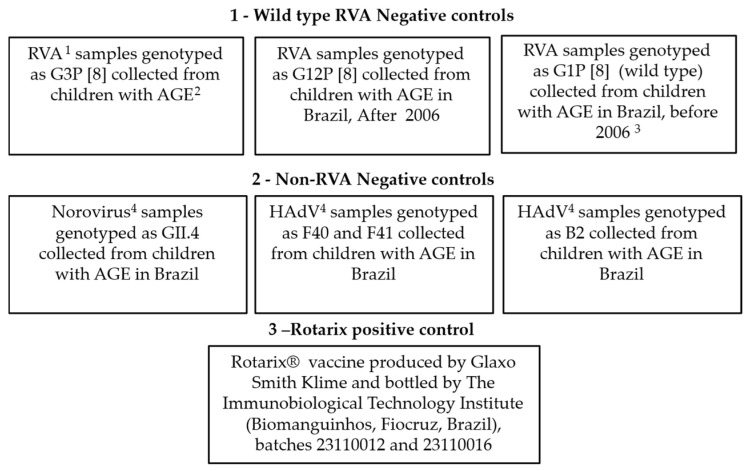
Negative and positive control samples stored in the LRRR Biobank used in qRT-PCR for the detection of vaccine-derived RVA G1P[8] [30]. These samples were used to validate the use of the described qRT-PCR by verifying specificity. ^1^ RVA = Rotavirus group A. ^2^ AGE = Acute gastroenteritis. ^3^ Samples characterized as vaccine (Rotarix^®^) and collected from children before the implementation of the vaccine (Rotarix^®^) by the Brazilian National Immunization Program (PNI), which occurred in March 2006. ^4^ HAdV = Human Adenovirus.

The negative control samples for initial internal validation of the qRT-PCR method by Gautam et al., used to distinguish wild-type RVA G1P[8] samples from those derived from the vaccine (Rotarix^®^), were fecal samples stored in the LVCA-RRRL Biobank and are described in Figure 1. Fecal samples are systematically sent through sentinel sites in the State Central Laboratories. Positive control samples were Rotarix^®^ vaccines produced by GlaxoSmithKline and bottled by the Immunobiological Technology Institute (Oswaldo Cruz Foundation).

### 2.3. Extraction of Total Nucleic Acids and qRT-PCR to Detect Rotavirus

Total nucleic acids were obtained from 200 µL of 10% fecal suspensions (samples collected in 2021 and negative controls) and Rotarix^®^ vaccine (Figure 1). The automated total nucleic acid extraction procedure was performed using the TANBead Maelstrom™ 9600 system (Taiwan Advanced Nanotech, Taoyuan, Taiwan) and the Extract^®^ DNA and RNA of Pathogens MDx kit (Loccus^®^), following the manufacturer’s instructions, with a final eluted sample volume of 60 µL. The isolated total viral nucleic acid was immediately stored at −80 °C until molecular analysis. For samples collected in 2021, monoplex qRT-PCR was performed for RVA detection as previously described [5]. All samples that showed signals crossing the threshold line in both replicas up to a cycle threshold (Ct) value of 38.00 and presented a characteristic sigmoid curve were regarded as positive.

### 2.4. Specific qRT-PCR to Distinguish Wild-Type RVA G1P[8] Samples from Those Derived from Vaccine (Rotarix^®^)

An initial test using only total nucleic acids from the control sampling (Figure 1) was performed for internal validation and to establish specific qRT-PCR to distinguish wild-type RVA G1P[8] samples from those derived from the vaccine (Rotarix^®^) [30]. Subsequently, 158 other total nucleic acids (sampling) from fecal samples collected in 2016, 2017, and 2021 from children residing in the Amazon region were subjected to specific qRT-PCR analysis. Between the years 2016 and 2017, 138 samples were previously positive for RVA [5]. Twenty samples were collected in 2021 and tested positive for RVA in this study (Section 2.3). The primers and probes (FastBio Ltd., São Paulo, SP, Brazil) used are described in Table 1.

The positive and negative control samples (Figure 1), as well as the samples collected from children in the Amazon region, were submitted for the three primer and probe pairs separately (Table 1) in three monoplex qRT-PCR assays. All reactions were performed on the QuantStudio™ 3 Real-Time PCR System (Applied Biosystems, Foster City, CA, USA) using the SuperScript™ III Platinum™ One-Step qRT-PCR kit (Thermo Fisher Scientific, Waltham, MA, USA), according to the manufacturer’s recommendations, in a total volume of 20 mL and under optimized thermal cycling conditions, as follows: A reverse transcription cycle at 50 °C for 15 min, followed by initial denaturation at 95 °C for 2 min, then 45 cycles at 95 °C for 15 s and 60 °C for 1 min. All samples were evaluated in duplicate, and those that showed signals crossing the threshold line in both replicates up to a Ct value of up to 43.00 and exhibited a characteristic sigmoid curve were considered positive.

### 2.5. PCR Amplification and Nucleotide Sequencing of the VP4 (VP8*) and VP7 Genes of Vaccine (Rotarix^®^) Samples

Samples characterized as G1P[8] derived from vaccine (Rotarix^®^) after specific qRT-PCR testing (Section 2.4) were subjected to PCR amplification of the RVA VP4 (VP8*) gene. These samples presented low viral load, and due to the difficulty of amplification, a PCR protocol for RVA was developed. Synthesis of cDNA was performed with 12.5 µL of 10X RT random primers from the High-Capacity cDNA Reverse Transcription kit (Thermo Fisher Scientific) added to 11.5 µL of RNA extracted in Section 2.3. The random primers and RNA extracted were hybridized at 97 °C for 5 min, followed by incubation for 3 min on ice. The SuperScript™ III One-Step RT-PCR system with Platinum™ Taq DNA kit was used (Thermo Fisher Scientific), and 25 µL of the 2X reaction mixture with 1 µL of the SuperScript™ III RT/Platinum™ Taq Mix enzyme (Thermo Fisher Scientific) was added to the tube containing the random primers and the previously hybridized extracted RNA. The reaction was incubated at 50 °C for 30 min and then at 95 °C for 5 min. A total of 2.5 µL of cDNAs was subsequently used for single-cycle PCR reactions in a final volume of 25 µL using iTaq (BioRad Laboratories, Hercules, CA, USA), according to the manufacturer’s instructions. The cycling programs consisted of initial denaturation at 94 °C for 10 min, followed by 40 cycles of 94 °C for 30 s, 49 °C for 30 s, 72 °C for 1 min and 30 s, and a final extension at 72 °C for 7 min. VP4F/VP4R (663 pb) primers were used at a concentration of 20 mM [33]. The cDNA obtained above was also used to exceptionally amplify the VP7 gene, and the same PCR protocol described above was used, except for the primers, which were VP7F/VP7R (884 bp) [34].

For Sanger sequencing, the amplified samples were purified using the Wizard^®^ SV Gel kit and a PCR Clean-Up System (Promega, Madison, WI, USA), following the manufacturer’s instructions. The purified amplified samples of the VP4 (VP8*) and VP7 genes from RVA were analyzed by Sanger sequencing using the BigDye^®^ Terminator v3.1 sequencing kit and the ABI Prism 3730^®^ genetic analyzer (Applied Biosystems, Foster City, CA, USA), with the same primers used in the PCR method, through the Oswaldo Cruz Institute (Fiocruz) Sanger sequencing platform.

### 2.6. Phylogenetic Trees, G1P[8] Designation, and Statistical Analysis

The chromatograms of the G1P[8] derived from the vaccine (Rotarix^®^) were analyzed using the free trace viewer Chromas 2.4 (Technelysium Pty Ltd., South Brisbane, QL, Australia). RVA nucleotide alignment was performed using Mega Molecular Evolutionary Genetic Analysis software version 12.1, comparing with reference nucleotide sequences available from the National Center for Biotechnology Information (NCBI); for VP4 (VP8*), accession numbers JX406750.2 (G1P[8], Wa, wild type), JX943612.2 (G1P[8], Rotarix^®^ vaccine), and MZ209043.1 and ON736925.1 (G3P[8], wild type) were used. For VP7, MT025872.1 (G1P[8], Wa, wild type), JX943614.2 (G1P[8], Rotarix^®^ vaccine), and OR675129.1 and AY707792.1 (G3, wild type) were used. To confirm the G1P[8] vaccine samples, the Rotavirus Genotyping Tool Version 0.1 was used (https://www.rivm.nl/mpf/typingtool/rotavirusa/how-to-use, accessed on 30 November 2025). For statistical analyses, when necessary, the R software (version 4.5.1) was used.

### 2.7. In Silico Structural Modeling and Docking of the VP4 Protein (VP8* Domain) with HBGA Sugars

The VP8* domains of the G1P[8] (Rotarix^®^ vaccine) reference JX943612.2 (protein ID: AGA83295.2), the G1P[8] Wa reference JX406750.2 (protein ID: AGJ72856.1), G1P[8] samples, the G3P[8] Wa reference MN366044.1 (protein ID: QIN53370.1), and G3P[8] samples were modeled to allow structural comparisons. The models were generated independently using Phyre2.2 (https://www.sbg.bio.ic.ac.uk/phyre2/html/page.cgi?id=index, accessed on 25 November 2025), where they were produced in Intensive mode. The resulting structures from the Protein Data Bank (PDB) were evaluated for structural quality, Vina score, and cavity volume. A high-confidence model for each sample was selected for molecular docking assays where two HBGA sugars were evaluated: type 1 H antigen (PubChem CID 5326971), corresponding to the trisaccharide Fuc(α1-2) Gal(β1-3) GlcNAc, and its precursor LNB (PubChem CID 440994), corresponding to the disaccharide Gal(β1-3)GlcNAc. The linking sugars were obtained directly from PubChem in structural data file (SDF) format using the available 3D files. Molecular docking assays were performed using CB-Dock2 (http://183.56.231.194:8001/cb-dock2/php/blinddock.php, accessed on 30 November 2025). The PDB files of the VP4 (VP8*) proteins and the SDFs of the ligands were loaded directly onto the platform. CB-Dock2 automatically detected five potential cavities and performed calculations using AutoDock Vina 1.2.0.

#### Structural Refinement of Protein–Sugar Interactions and Estimation of Atomic Proximity at the Binding Site

For refinement and identification of physically plausible interactions, protein–sugar–ligand contact analysis was performed in PyMOL v.2.5 (Schrödinger LLC) [35]. Hydrogen atoms were removed to minimize uncertainties related to their structural modeling [36,37]. The analysis considered exclusively heavy atoms, and the ligand was defined using the organic selection criterion. Residual non-polypeptide components present in the PDB files were removed to avoid non-biological signaling in the VP4 (VP8*) protein delimited as a polymer. Only residues that presented at least one heavy atom positioned ≤ 4.0 Å from a heavy atom of the ligand were considered direct contacts. The 4.0 Å cutoff parameter was considered a threshold for capturing short-range van der Waals contacts and possible hydrogen-mediated interactions, excluding artifacts resulting from a wide spatial neighborhood [38,39]. The ångström (Å), equivalent to 10^−10^ m, is the standard scale for structural measurements at the atomic level. Contact residues were identified in PyMOL using the following commands: remove elem H; select link, organic; select prot, polymer and not elem H and not lig; select prot_near_lig, prot within 4.0 of lig; iterate (prot_near_lig and CA name), print (string, resi, resn). All iterations were visually inspected for topological confirmation and elaboration of the final graphical representations.

### 2.8. Secretor Status and Lewis Antigen Phenotyping

The HBGA phenotyping for samples collected in 2021 was performed using the processed saliva samples diluted 1:100 via Enzyme Immunoassay (EIA), as previously described, to detect A, B, Lea, and Leb antigens [40,41]. Additionally, all saliva samples diluted 1:100 were assayed for Fucα1-2Gal-R detection using the lectin-based EIA [40,41] to confirm the secretor phenotype. All saliva samples were evaluated in duplicate together with control saliva samples from secretor and non-secretor adult donors (Ethics Committee of the Oswaldo Cruz Foundation, Fiocruz, Brazil; Approval number 94144918.3.0000.5248, on 20 August 2018).

## 3. Results

### 3.1. Low Frequency of RVA Detection During the COVID-19 Pandemic in Children Living in the Amazon Region

The frequency of RVA detection by qRT-PCR (Section 2.3) in the fecal samples collected from children living in the Amazon region in 2021 was 11.4% (23/202). About half of the children presented with AGE, with 12.9% (13/101) tested positive for RVA; in children without AGE (control), RVA was detected in 15.8% (16/101) of the cases. The median Ct value detected by qRT-PCR for RVA was 34.8 (lowest value 23.5, highest value 38.5). The HBGA (Secretor and Lewis) profile for samples from 2021 was as follows: Secretor children comprised 93.1% (188/202), with 76.6% (144/188) being Lea-Leb+/Lea+Leb+ and 23.4% (44/188) being Lea-Leb-. Non-secretor children represented 6.9% (14/202), with 78.6% (11/14) being Lea-Leb- and 21.4% (3/14) Lea+Leb-.

### 3.2. The Specific qRT-PCR Distinguished Wild-Type RVA, Human Adenovirus, and Norovirus Controls from Vaccine (Rotarix^®^)

**Figure 2:**
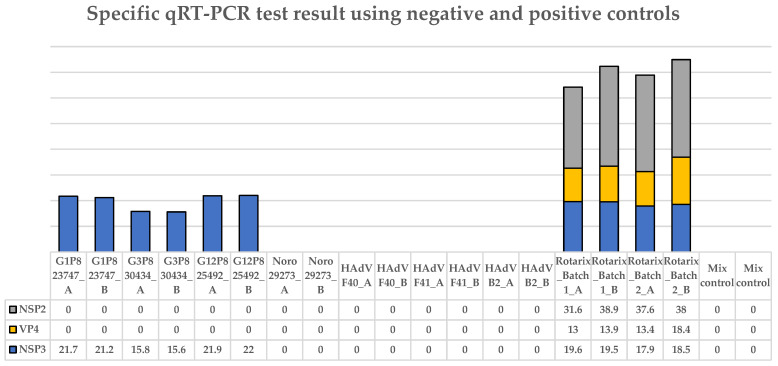
Results of the qRT-PCR specificity verification for the detection of the Rotarix^®^ vaccine using negative and positive controls identified in the table below the graph. Each control tested is identified by the virus name, number, and/or genotype. For each gene tested (NSP3, VP4, and NSP2), the Ct values are also presented in the table. Positivity for the NSP3, VP4, and NSP2 genes is indicated in the graph by blue, yellow, and/or gray bars. All samples were tested in duplicate, indicated by the letters A or B.

The qRT-PCR method by Gautam et al. specifically distinguished wild-type RVA G1P[8], G3P[8], and G12P[8] fecal samples from the Rotarix^®^ vaccine. No cross-reaction was observed with other viral strains causing AGE (norovirus and HAdVs F40, 41, and B2). The Rotarix^®^ vaccine strains (two different batches) showed positivity, with a low Ct value (Figure 2). The Ct values presented for the NSP3 and VP4 genes were considerably lower than for the NSP2 gene when the same control sample, containing approximately the same number of molecules, was used. All positive control samples showed characteristic sigmoid curves for the three genes, consistent with positive samples.

### 3.3. Children Not Vaccinated with the Rotarix^®^ Vaccine Who Live in the Amazon Region Eliminate Rotarix^®^ G1P[8] Vaccine

The frequency of samples collected in 2016, 2017, and 2021 from children residing in the Amazon region that were considered positive for the specific qRT-PCR detection of the Rotarix^®^ vaccine was 18.3% (29/158). A sample was considered positive if it was positive for the NSP3 gene of the control RVA and for at least one of the two genes, with specific primers (VP4 and NSP2). The median Ct value detected by qRT-PCR for the NSP3, VP4, and NSP2 genes were, respectively, 35.3 (minimum value of 27.1 and maximum value of 40.4); 38.9 (minimum value of 31.4 and maximum value of 42.8), and 38.0 (minimum value of 34.6 and maximum value of 42.0).

**Figure 3:**
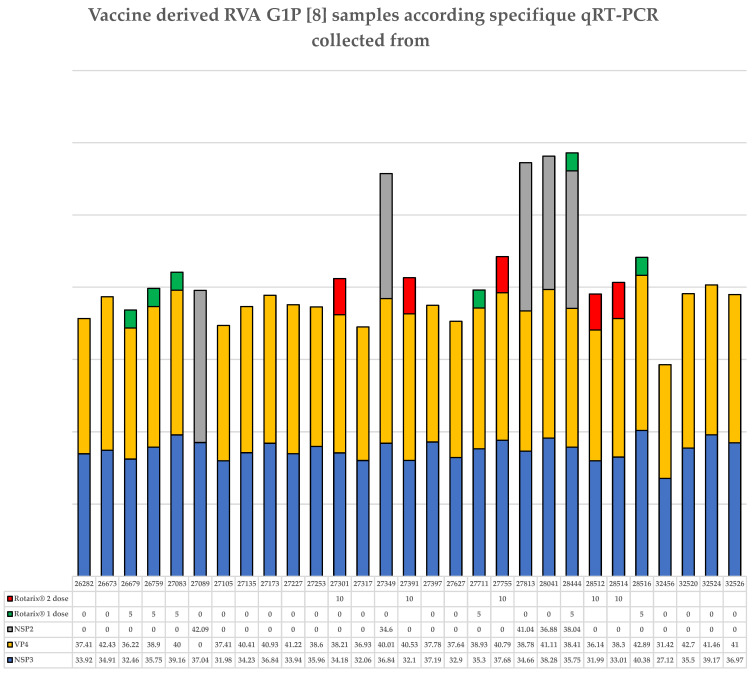
Detection profile of samples identified as RVA G1P[8] derived from the Rotarix^®^ vaccine collected from fecal shedding of children living in the Amazon region. Each sample tested is presented by its identification number (ID). For each gene tested (NSP3, VP4, and NSP2), the Ct values are also presented in the table. Positivity for the NSP3, VP4, and NSP2 genes is indicated in the graph by blue, yellow, and/or gray bars. Samples with green and red bars were collected from children who received one (value number 10) or two doses (value number 20) of the Rotarix^®^ vaccine, respectively.

The positivity for the NSP3, VP4, and NSP2 genes of RVA and the Rotarix^®^ vaccination profile for each sample collected from children residing in the Amazon region infected with the Rotarix^®^ vaccine are shown in Figure 3.

In total, 100% (29/29) of the samples characterized as the Rotarix^®^ vaccine repeated positivity for the NSP3 gene (control) with Ct values very close to the initial RVA detection test, as described previously for the 2016 and 2017 samples [5] and for the 2021 samples (Section 3.1). A total of 96.5% (28/29) of the samples were positive for the VP4 gene, and only 17.4% of the samples (5/29) showed positivity for the NSP2 gene. All samples positive for NSP2 were also positive for the VP4 gene, except for sample 27089, which showed positivity only for the NSP2 gene (Figure 3). Eighteen of the 29 samples characterized as the Rotarix^®^ vaccine (62%) were collected from children who had not received any dose of the Rotarix^®^ vaccine, corresponding to 11.4% (18/158) of the total samples collected and tested for the detection of the Rotarix^®^ vaccine. Six children (20.7%; 6/29) received one dose of the Rotarix^®^ vaccine, and five (17.2%; 5/29) received two doses. All children who received one or two doses of the Rotarix^®^ vaccine showed detection only for the VP4 gene, except for sample 28444, which showed detection for the NSP2 gene.

All samples characterized as the Rotarix^®^ vaccine were collected from children residing in Brazil in different municipalities of the states of Roraima (*n* = 24) and Amazonas (*n* = 3), including isolated regions of the Amazon rainforest. Two samples (*n* = 2) were collected from children residing in Venezuela. A total of 75.8% (22/29) of the children presented AGE, and of these, 68.2% (15/22) had not received the Rotarix^®^ vaccine. Three of the four samples positive for the VP4 and NSP2 genes were collected from children who had also not received Rotarix^®^ (Table 2). The HBGA (secretory and Lewis) profiles of samples collected from children with the Rotarix^®^ vaccine detection are presented in Table 2. All non-secretory children (*n* = 6) presented with AGE, and all those without AGE (*n* = 7) were secretors. A total of 79.3% (23/29) of the samples characterized as the Rotarix^®^ vaccine were collected from secretor children. Although non-secretor children were exclusively observed among AGE cases and all asymptomatic children (control) were secretors, this association was not statistically significant (Fisher’s exact test, *p* = 0.29).

### 3.4. The VP4 (VP8*) Gene of the Rotarix^®^ Vaccine Shedding from Children Living in the Amazon Region Exhibits Point Mutations

Ten samples detected as the Rotarix^®^ vaccine were considered eligible for PCR amplification after specific qRT-PCR. These samples presented low viral loads, but the PCR protocol described in Section 2.5 was efficient in their amplification, resulting in good quality amplicons suitable for nucleotide sequencing by the Sanger method. Analysis of the nucleotide sequences of the 10 samples confirmed that 8 were the Rotarix^®^ vaccine G1P[8]. Two samples were characterized as G3P[8]. Mutations were detected in all 10 samples, when compared with the consensus including the reference sequences available in the NCBI GenBank, as described in Section 2.6.

**Figure 4:**
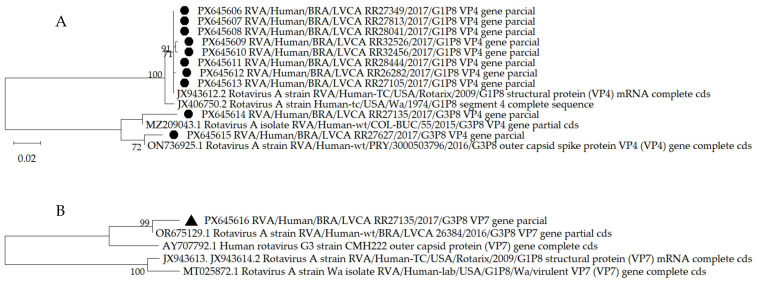
Phylogenetic analyses of the nucleotide (nt) sequences of positive samples identified as the Rotarix^®^ vaccine by specific qRT-PCR collected from fecal shedding of children living in the Amazon region (marked with a black-filled circle). (**A**) = Structural protein (VPs) VP4 (VP8*; 658 nt) and (**B**) = VP7 (842 nt). Reference RVA strains from GenBank labeled were described (Section 2.6). Maximum-likelihood phylogenetic trees were constructed with MEGA 12 software and bootstrap tests (1000 replicates) based on the Tamura three-parameter (VP4) and Tamura-Nei + I model. Bootstrap values above 70% are given at branch nodes.

Few mutations were detected in the Rotarix^®^ vaccine samples in the VP4 gene (VP8*), these being one silent mutation at position 144C > G (ID 26282) and three missense mutations at positions 499T > C (amino acid change F167L; samples ID 32456 and 32526); 644G > C (C215S; sample ID 32526) and 787G > A (E263K; sample 32456) (Table S1, Supplementary Material). All samples produced by missense mutations were collected in 2021. Two samples were, respectively, characterized as G3P[8] (ID 27135) and G3P[8] candidate (ID 27627), despite being detected in the vaccine-specific qRT-PCR (both positive only for the VP4 gene), and showed, respectively, similarity with G3P[8] equine-Like G3P[8] from a child with GA in Colombia collected in 2015 (accession number MZ209043.1) and with G3P[8] collected in 2016 in Paraguay (accession number ON736925.1) (Figure 4A).

The nucleotide sequence of the VP7 gene from sample 27135 was obtained and confirmed as G3P[8] using BLAST 2.17.0 and Rotavirus Genotyping tools version 0.1. Sample 27627 was not eligible for VP7 gene amplification by PCR. Phylogenetic analysis showed similarity of the 27135 sample with G3P[8] collected in Brazil in 2016 (MH569776.1), being in the same clade as sample G3 (AY707792.1) detected in a child with AGE in 2000 in Thailand during a G9 outbreak at a children’s hospital [42] (Figure 4B).

### 3.5. In Silico Analysis of the Mutations Showed No Impact on the Protein Structure of VP8* Rotarix^®^ Mutants

**Figure 5:**
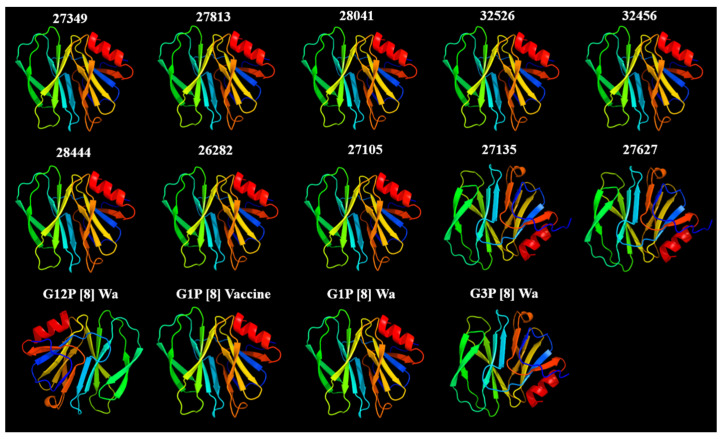
In silico structural models based on VP4 protein homology (VP8* domain) were generated using Phyre2.2. Ribbon representations of the VP4 protein homology models (VP8* domain) generated for all samples detected in the specific Rotarix^®^ vaccine qRT-PCR were analyzed using Phyre2.2 in Intensive mode. In addition to the samples (IDs indicated), the Rotarix^®^ G1P[8] and wild-type G1P[8] Wa, G3P[8], and G12P[8] were included in the analysis and model construction. For each sample, the structural model with the highest confidence was selected based on the Phyre2.2 confidence score, alignment coverage, and structural quality metrics.

In silico structural models were constructed considering the nucleotide sequences obtained and deduced for the VP4 (VP8*) protein from fecal shedding and characterized as the Rotarix^®^ vaccine from children residing in the Amazon region (Figure 5). To compare the structure of the VP4 (VP8*) protein, reference samples indicated in Figure 5 were also constructed. The predicted models showed high confidence values (Phyre2.2 confidence ≥ 90–100%) and good alignment coverage, confirming their suitability for subsequent functional analyses. All structures exhibited the conserved β-sandwich fold characteristic of the VP8* lectin-like domain, composed of mixed elements of β chains and α helices. The predicted models were consistent with the nucleotide homology classification. Samples ID 27135 and 27627 fit a fold similar to the pattern predicted by the G3P[8] reference nucleotide sequence. The same was observed for samples characterized by specific qRT-PCR as the Rotarix^®^ G1P8 vaccine. No difference was observed in the folding between VP4 (VP8*) G1P[8] and wild-type G1P[8].

### 3.6. In Silico Interactions of the VP4 (VP8*) Protein from the Rotarix G1P Vaccine [8] and the Wild Type with the Sugars H1- and Lacto-N-Biose Are Similar

**Figure 6:**
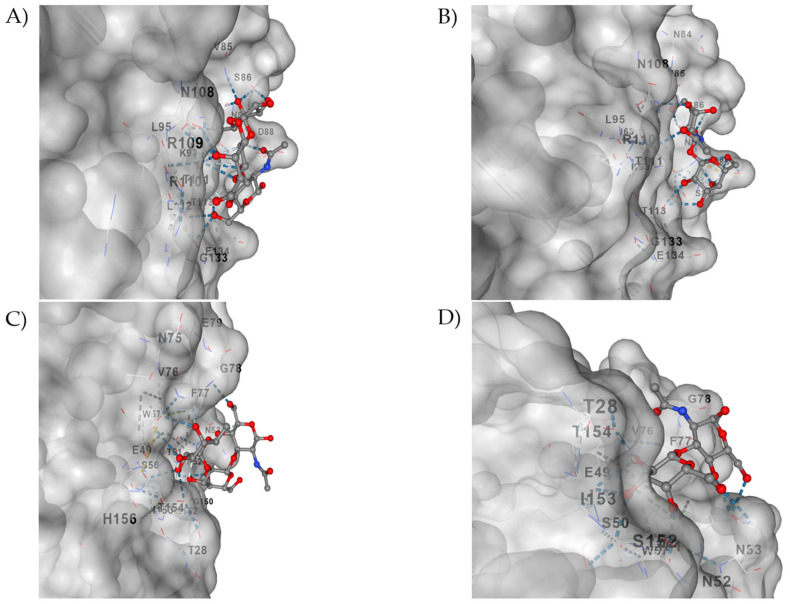
In silico docking interactions of VP4 (VP8* domains) from RVA reference sample genotypes G1P[8] and G3P[8] with H1-type sugars (Fucose) and LNB (Precursor). The figure provides a detailed view of the sugar-binding site with VP8*. Figures (**A**) and (**B**) show, respectively, the docking of the VP8*/G1P[8] genotype with H1-type sugar (CID: 5326971) and LNB (CID: 440994). Figures (**C**,**D**) show the same type of docking using VP8* from the G3P[8] genotype. All sugar ligands were obtained in SDF format from PubChem and coupled using CB-Dock2.

**Figure 7:**
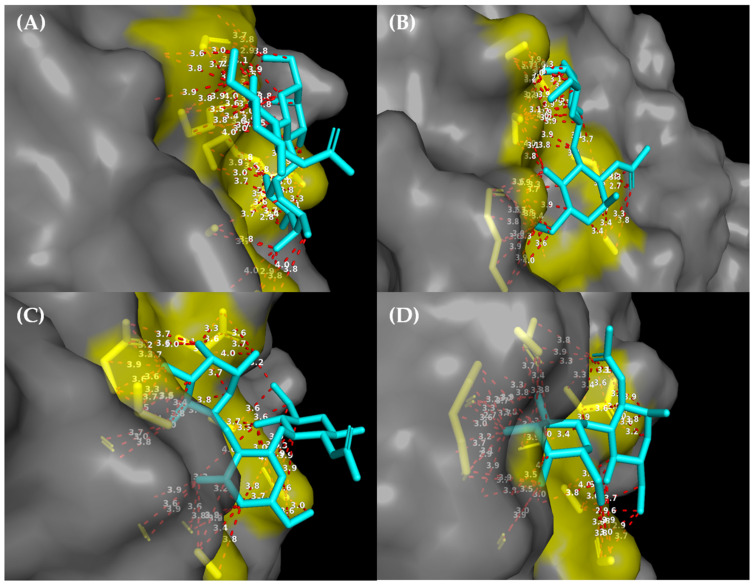
Structural mapping of direct interactions between VP4 (VP8*) genotypes G1P[8] and G3P[8] and HBGA sugars. Surface visualization of the VP4 (VP8*) domain highlighting all heavy-atom contacts (≤4.0 Å) formed with each evaluated sugar. The protein is displayed as a semi-transparent gray surface, with directly interacting residues represented as yellow sticks, while the bound sugars H type 1 and LNB appear as cyan sticks. Red dashed lines indicate all interatomic contacts within the cutoff threshold, with distance labels shown in ångströms (Å). Only residues meeting the spatial criterion were retained to ensure depiction of direct molecular recognition. Structural analysis and visualization were performed using PyMOL v.2.5 (Schrödinger LLC). Panels: (**A**) VP8* from G1P[8] complexed with H type 1; (**B**) VP8* from G1P[8] complexed with LNB; (**C**) VP8* from G3P[8] complexed with H type 1; (**D**) VP8* from G3P[8] complexed with LNB.

Predicted sites in silico models by docking were obtained for the VP4 (VP8*) protein of the G1P[8] and G3P[8] genotypes (Figure 6). With the purpose of functionally validating the sites, a structural analysis based on spatial proximity was performed to identify exclusively direct interactions between VP4 (VP8*) and the evaluated sugars (Figure 7). Only heavy atoms of the protein located at ≤4.0 Å from the ligands (sugars) were considered as contacts, allowing the detection of relevant interactions, such as potential hydrogen bonds and hydrophobic contacts, while residues distant from the interaction interface were excluded. This approach revealed significant differences among the analyzed complexes. In the VP4 (VP8*) of G1P[8], the H type 1 antigen established direct contact with three residues (ARG109, SER143, and SER144), whereas LNB interacted only with two residues (VAL85 and SER86). In contrast, for the VP4 (VP8*) of G3P[8], H type 1 presented only one residue in direct contact (PHE77), constituting the scenario of the lowest structural engagement observed. In contrast, LNB formed three contacts with the VP4 (VP8*) of G3P[8] (SER50, PHE77, and ILE153), suggesting an alternative accommodation of this sugar in the protein interface. This data highlights that the affinity and specificity of interaction of VP4 (VP8*) vary according to the viral genotype and the degree of structural complexity of the ligand.

## 4. Discussion

A high frequency of RVA circulation has been detected in this study’s cohort from the Amazon region. The frequency detected in 2016/2017 was 21.38% [5], and in the study presented here, with samples collected in 2021 (COVID-19 pandemic period), 11.4% (23/202) of RVA positivity was detected, and half of the children presented with AGE (12.9%; 13/101). The lower frequency detected in 2021 was still high compared to that detected in other Brazilian states in the same period [43]. Brazil is a region with a large population, and although the Brazilian National Immunization Program (PNI) excels in the distribution of the Rotarix^®^ vaccine, the low coverage in the Amazon region [5] certainly promotes the maintenance of RVA in that population. Of the total positive RVA samples in the years 2016, 2017, and 2021, the frequency of the G1P[8] vaccine genotype detected via testing with a qRT-PCR specific for vaccine (Rotarix) G1P[8] was 18.3% (29/158). One sample was genotyped for the VP7 gene and confirmed to be G3P[8], and another sample was considered a G3P[8] candidate due to the lack of G (VP7) genotyping in accordance with the low viral load.

The samples tested in the specific qRT-PCR presented a low viral load (median Ct value of 35.3) for the RVA NSP3 control gene. Viral shedding of the Rotarix^®^ vaccine occurs within a maximum of 30 days [44]. The simultaneous detection of the VP4 and NSP2 genes may have been affected. [30] described that, using a series of 10-fold serial dilutions of the dsRNA template derived from the Rotarix^®^ NSP2 vaccine, the efficiency of the Rotarix^®^ NSP2 assay was calculated at 94%, with a detection limit of two copies. For the VP4 gene, using the same diluted template, the efficiency of the Rotarix^®^ VP4 assay was calculated at 105%, with a detection limit of three copies. Therefore, the sensitivity of the test described by Gautam et al. is higher for the VP4 gene.

The low viral load of the identified G1P[8] Rotarix^®^ vaccine samples impacted the amplification of VP4 gene. Indeed, the median Ct value for these samples was high for the NSP3 gene (35.3). The Ct values detected for the VP4 and NSP2 genes for the Rotarix^®^ vaccine were even higher (low viral load). We hypothesized that the two samples genotyped by conventional PCR (VP4/VP7 genes) as G3P[8] (ID 27135) and G3P[8] candidate (ID 27627) show successful amplification because they represent a mixed genotype population, and the G1P[8] genomes were not eligible for amplification due to the low viral load. Wild-type RVA genotypes mixed with vaccine samples were detected by Gurgel et al. [45]. The hypotheses corroborate the difficulty of amplifying the Rotarix^®^ vaccine genomes and demonstrate the sensitivity of specific qRT-PCR with primers specifically designed to amplify Rotarix^®^ vaccine. The specificity of the specific qRT-PCR to detect Rotarix^®^ vaccine was verified using wild-type G3P[8] samples that did not amplify in the validation test. G3P[8] VP4 PCR amplified samples are in fact G3P[8], according to the in silico model deduced from the VP4 nucleotide sequences, which will be discussed below. A total of 75.8% (22/29) of the children presented with AGE, but we cannot disregard the pathogenic agents causing AGE associated with the etiology of AGE, mainly noroviruses. In fact, in the study that preceded this one, noroviruses could be statistically considered the main etiological agent of AGE [5]. Although some children in this cohort were hospitalized, none died, ruling out a severe case associated with Rotarix^®^ attenuation reversal. It would be interesting to apply the specific qRT-PCR test revisited here by us with success [30] for the large percentage of RVA G1P[8] samples, which has still been detected in high frequency in samples from around the world [46]; this may be vaccine-related because of good vaccination coverage or indirect protection from Rotarix^®^.

In this study, we highlight the possible occurrence of indirect protection, also called herd immunity. The Rotarix^®^ vaccine, being oral and potentially conferring indirect protection, is especially useful in densely populated areas or where access is difficult, such as the Amazon region. Transmission of the rotavirus vaccine or recombinant strains of the vaccine to unvaccinated contacts has already been reported, as reviewed [47]. The potential for indirect protection verified in our study was 11.4% (18/158). Verification of Rotarix^®^ IgA antibody titers associated with these samples would be necessary to confirm indirect protection. Interestingly, RVA was detected more frequently in children without AGE (15.8%) compared with children with AGE (12.9%), and we could also associate this result with the possibility of indirect protection occurring in non-AGE children, since RVA was not the main causative agent of AGE in this cohort, as previously presented [5].

The secretor profile of children in Brazil has been presented and is predominantly secretor [5,40,44,48]. To confirm this data, the samples collected from this study in 2021 were predominantly secretor (93.1%; 188/202). A larger number of non-secretor children would be needed to confidently state that secretor children had more Rotarix^®^ G1P[8] shedding because the vaccine has greater avidity for its HBGA receptor [27]. In this context, we journeyed through in silico protein models. These models were deduced from the 10 sequences that were characterized by a specific qRT-PCR Rotarix^®^ G1P[8], along with G1P[8] wild-type, G3P[8], and G12P[8] genotype reference samples. The models confirmed the sequencing data and the eight G1P[8] Rotarix^®^ samples, which are structurally identical to the Rotarix^®^ vaccine G1P[8]. The amino acid changes detected, including the F167L amino acid change described as attenuation reversal [44,49], did not affect the in silico constructed structures of the VP4 (VP8*) samples in this study characterized as Rotarix^®^ G1P[8], confirming previous findings [49]. However, the impact of HBGA secretor status on changes in amino acid F167L has been discussed [50].

In this study, in silico analysis of VP4 (VP8*) binding to the two sugars that define HBGA secretor status (H1 and LNB) is presented. The VP4 [VP8*] proteins were deduced from reference nucleotide sequences of the G1P[8] and G3P[8] genotypes. Our structural findings show that the ability of VP4 (VP8*) to recognize sugars varies for the two genotypes selected here according to the type of sugar (H1 or LNB). In fact, VP4 (VP8*) plays an essential role in adhesion to cell surface sugars (HBGAs or sialic acid) depending on the strain [18,51,52,53]. Studies with human sample VP4 (VP8*) genotypes P[8] refs. [4,6] indicate the recognition of HBGAs as a susceptibility factor to the virus [18,54,55]. In our study, the G1P[8] + H type 1 complex presented three direct contact residues (ARG109, SER143, SER144), suggesting a relatively stable and compatible interface with considerable affinity. This profile reinforces that fucosylated HBGAs (H1) are preferential ligands for P[8] variants, corroborating previous data [55,56]. However, the G1P[8] + LNB complex presented only two direct contacts (VAL85, SER86), indicating weaker affinity for this non-fucosylated precursor. This is consistent with the observation that terminal fucose, present in H1, can increase interaction stability through additional and specific contacts [18,49,56]. Interestingly, G3P[8] behaves differently from G1P[8] with H1 in direct contact with only one residue (PHE77), suggesting that this variant would have reduced structural affinity for this sugar. With LNB, there were three contacts (SER50, PHE77, ILE153), indicating that G3P[8] could accommodate less complex or non-fucosylated sugars. This result suggests an adaptive flexibility of the G3P[8] binding site, which may reflect a differentiation in the tropism or repertoire of receptors used/investigated by this genotype [18,57,58].

The RVA G3P[8] genotype has been prevalent in low- and middle-income countries, including Brazil [46]. In this study, we describe the detection of two samples identified by specific qRT-PCR as vaccine-related (Rotarix^®^). The sample confirmed as G3P[8] by sequencing the VP7 gene was collected in March 2017 (ID 27135) when the equine-like RVA G3P[8] genotype appeared in Brazil. Sample ID 27135 showed 97.5% similarity to a non-equine-like G3P[8] sample collected in 2015 in Colombia from a child with AGE (VP4 gene) and 100% similarity to the equine-like G3P[8] sample collected in 2016 during the dissemination of this genotype in Brazil [59]. Our hypothesis to be investigated is that the equine-like G3P[8] genotypes, detected initially in Paraná in 2015 and after in the northern region in 2016, spread rapidly throughout Brazil, with the contribution of state of Roraima (Amazon region). Studies with Rotarix^®^ vaccine samples derived from fecal shedding are challenging, mainly due to the low viral load and difficulty of RVA genome amplification. Here, we present molecular data from Rotarix^®^ vaccine samples derived from fecal shedding, which may contribute to understanding the evolutionary dynamics of rotavirus RVA. The limited sample size of those detected as Rotarix in this study, exclusively from children living in the northwestern Amazon region, is a limitation of our study and highlights the need for future studies applied involving larger-scale cohorts. As mentioned above, the detection of specific IgA antibodies to Rotarix could directly confirm immunological protection.

## 5. Conclusions

Here, we present molecular data from Rotarix^®^ vaccine samples obtained from fecal shedding, which may contribute to understand the evolutionary dynamics of RVA, reassuring its safety. The specific qRT-PCR method is a useful tool for molecular epidemiology studies and RVA surveillance. HBGA blood group antigens are highlighted as crucial in the infectious dynamics of RVA, according to the in silico models presented. The contribution of circulating genotypes in the state of Roraima (Amazon region) to RVA diversity will likely be investigated.

## Figures and Tables

**Table 1:** Primers and probes used in qRT-PCR to distinguish wild-type RVA G1P[8] samples from those derived from the vaccine (Rotarix^®^).

Primer (p) or Probe (pb) ID ^1, 2^	Nucleotide Sequence 5′-3′(Fluorophore/Quencher)	RVA Specificity/Gene ^3^	Reference
NSP3F (p)	ACCATCTWCACRTRACCCTCTATGAG	RVAWt ^3^/Rotarix/NSP3	[32]
NSP3R (p)	GGTCACATAACGCCCCTATAGC	RVAWt ^3^/Rotarix/NSP3	
NSP3RVA (pb)	AGTTAAAAGCTAACACTGTCAAA (VIC/BHQ)	RVAWt ^3^/Rotarix/NSP3	
NSP2F (p)	GAACTTCCTTGAATATAAGATCACACTGA	Rotarix^®^/NSP2	[30]
NSP2R (p)	TTGAAGACGTAAATGCATACCAATTC	Rotarix^®^/NSP2	
NSP2Rotarix^®^(pb)	TCCAATAGATTGAAGT {C} AGTAA “C” GTTTCCA (FAM/BHQ)	Rotarix^®^/NSP2	
VP4F (p)	TGTGAGTAA “C” GATTCAAATAAATGGAAGTT	Rotarix^®^/VP4	
VP4R (p)	TCACCATGAAATGTCCATACTCTTCCACCA	Rotarix^®^/VP4	
VP4F Rotarix^®^(pb)	ATA {C} CAGA {C} TTGTAGGAATA “Y” TTAAATA (FAM/BHQ)	Rotarix^®^/VP4	

^1^—ID = Identification. ^2^ Primers and probes according to Gautam et al. ^3^ = Gene corresponding to one of the 11 segments of RVA; Wt = wild type.

**Table 2:** Age, Rotarix^®^ vaccine profile, mutations detected in the VP4 gene, clinical information, and HBGA profile of each child, compared with the results obtained regarding positivity for the VP4 and NSP2 genes through specific qRT-PCR for detection of the Rotarix^®^ vaccine. Samples ID 27135 and 27627 shaded were, respectively, genotyped as G3P[8] (ID 27135) and G3P[8] candidate.

Sample ID	Child Age	Rotarix^® 1^ Dose = (0), (1) or (2)	RVA Rotarix^®^ Gene Detection	RVA VP4 (VP8*) Mutations (aa Change)	Clinical	HBGA ^2^ Sec+/Sec- (Lewis)
26282 ^3^	>6 months	0	VP4	144C > G (no change)	AGE	Sec+ (Lea+Leb+)
26673	>1 month	0	VP4		Control	Sec+ (Lea-Leb+)
26679	<3 months	1	VP4		AGE	Sec- ((Lea-Leb-)
26759	>3 months	1	VP4		AGE	Sec+ (Lea+Leb+)
27083	>3 months	1	VP4		Control	Sec+ (Lea+Leb+)
27089	>6 months	0	NSP2		AGE	Sec- (Lea-Leb-)
27105	>1 month	0	VP4		AGE	Sec+ (Lea-Leb+)
27135	<3 months	0	VP4		Control	Sec+ (Lea+Leb+)
27173	>6 months	0	VP4		AGE	Sec+ (Lea+Leb+)
27227	>3 months	0	VP4		AGE	Sec+ (Lea+Leb+)
27253	<3 months	0	VP4		AGE	Sec+ (Lea-Leb+)
27301	<3 months	2	VP4		AGE	Sec+ (Lea+Leb+)
27317	>3 months	0	VP4		AGE	Sec+ (Lea+Leb+)
27349	<3 months	0	VP4/NSP2		AGE	Sec- (Lea-Leb-)
27391	>1 month	2	VP4		AGE	Sec+ (Lea+Leb+)
27397	<3 months	0	VP4		AGE	Sec+ (Lea-Leb+)
27627	>3 months	0	VP4		AGE	Sec+ (Lea+Leb+)
27711	<3 months	1	VP4		AGE	Sec+ (Lea-Leb-)
27755	>6 months	2	VP4		AGE	Sec+ (Lea-Leb+)
27813	>6 months	0	VP4/NSP2		AGE	Sec+ (Lea-Leb+)
28041	>6 months	0	VP4/NSP2		AGE	Sec- (Lea-Leb-)
28444	>3 months	1	VP4/NSP2		Control	Sec+ (Lea+Leb+)
28512	>3 months	2	VP4		Control	Sec+ (Lea-Leb+)
28514	>6 months	2	VP4		AGE	Sec- (Lea-Leb-)
28516	<3 months	1	VP4		Control	Sec+ (Lea+Leb+)
32456	<3 months	0	VP4	499T > C (F167L) 787G > A (E263K)	AGE	Sec+ (Lea-Leb+)
32520	<3 months	0	VP4		AGE	Sec+ (Lea+Leb+)
32524	<3 months	0	VP4		AGE	Sec- (Lea-Leb-)
32526	>6 months	0	VP4	499T > C (F167L) 644G > C (C215S)	Control	Sec+ (Lea+Leb+)

^1^ = Number of doses of the Rotarix^®^ vaccine received by each child, where (0) is no doses; (1) is one dose, and (2) is two doses. ^2^ HBGA = Histo-Blood Group Antigen. ^3^ = Samples with identification numbers starting with the number “2” are samples collected in the years 2016/2017, where the HBGA results were previously described [5]. All samples starting with the number “3” were checked for the HBGA profile, as described in Section 2.8. The gray rows correspond to the identified RVA G3P[8] samples.

## Data Availability

RVA gene sequences obtained in the current study have been submitted to GenBank-NCBI under accession numbers PX645606 to PX645615 (VP4) and PX645616 (VP7).

## Supplementary Materials

The following supporting information can be downloaded at https://www.mdpi.com/article/10.3390/v18010070/s1. Table S1: Mutations detected in vaccine Rotarix^®^ samples in the VP4 gene (VP8*).

## Abbreviations

The following abbreviations are used in this manuscript:

AGE	Acute Gastroenteritis
Ct	Cycle Threshold
DOAJ	Directory of Open-Access Journals
dsRNA	Double-Stranded RNA
EIA	Enzyme Immunoassay
Fiocruz	Oswaldo Cruz Foundation
*FUT*2	Fucosyltransferase 2
HAdV	Human Adenovirus
HBGA	Histo-Blood Group Antigen
HCSA	Hospital da Criança de Santo Antônio
IOC	Instituto Oswaldo Cruz
LNB	Lacto-N-biose
LVCA-RRRL	Laboratory of Comparative and Environmental Virology–Regional Rotavirus Reference Laboratory
NCBI	National Center for Biotechnology Information
PCR	Polymerase Chain Reaction
PDB	Protein Data Bank
qRT-PCR	Quantitative Real-Time Reverse Transcription PCR
RV	Rotavirus
RVA	Rotavirus A
SDF	Structural Data File
TLA	Three-Letter Acronym
