# Supplementary material for: Examination of In Vivo Mutations in VP4 (VP8*) of the Rotarix® Vaccine from Shedding of Children Living in the Amazon Region
Source: Viruses. 2026 Jan 3;18(1):70. doi: 10.3390/v18010070 (PMC12846572; doi:10.3390/v18010070)
Supplement: Supplementary file 1 [file viruses-18-00070-s001.zip › viruses-4045225-supplementary.pdf]

Table S1 Supplementary Material: Mutations detected in vaccine Rotarix® samples in the VP4 gene (VP8*)

Nt[1] Position (AA)	(Consensus) Rotarix® (JX943612.2)[2]	Sample ID										G1P [8] Wt (JX406750.2)	G3P [8] (MN366044.1)
		26282	27105	27135	27349	27627	27813	28041	28444	32456	32526		
144 (V)	C	G				G(G)							
152 (D)	A											T(V)	G(G)
162(E)	G												A
174(S)	G												A
177(T)	T												C
183(V)	A					G							G
210(Q)	G					A							A
213(P)	A					T							T
225(T)	T			A		A							A
233(N)	A			C(T)		C(T)							C(T)
255(N)	T			C		C							C
279(Y)	T			C		C							C
282(E)	A			G		G							G
300(D)	C					T							T
316(V)	G			A(I)		A(I)							A(I)
321(A)	T			A									
322(I)	A					G(V)							G(V)
336(V)	A											T	
337(D)	A					G(D)							G(D)
339(N)	C			T		T(D)							T(D)
359(M)	T			A(N)		A(N)						C(T)	A(N)
360(M)	G			T(N)		T(N)						(T)	T(N)
361(I)	A			G(V)									G(V)
374(S)	G			A(N)		A(N)							A(N)
375(S)	C			T(N)		T(N)						T	T(N)
378(K)	G			A		A							A
384(F)	T												C
390(V)	G			A		A							A
393(S)	T			A(R)		A(R)							A(R)
396(N)	C			T		T							T
403(N)	A			G(D)		G(D)							G(D)
414(K)	G			A									
418(L)	T					C							C
433(S)	A			G(G)		G(G)							G(G)
446(N)	A					G(S)							G(S)
450(E)	A			C(D)		C(D)							C(D)
465(H)	T			C									C
469(L)	T			C		C							
483(T)	C			T		T						T	T
485(R)	G			A(K)		A(K)							A(K)
489(L)	T			C		C							C
492(V)	A			G									
499(F)	T									C(L)	C(L)		
501(F)	T			A(L)		A(L)						G(L)	A(L)
516(R)	A					G							
517(V)	G			A(I)		A(I)							A(I)
543(P)	G												
546(R)	A												
552(T)	T			C									
555(T)	T					C							C
558(D)	C			T		T							T
561(S)	T					C							
566(N)	G			A(N)		A(N)							A(N)
567(S)	T			C(N)		C(N)							C(N)
571(A)	G												A(T)
579(L)	A			G		G							G
582(N)	T			C(G)		C(G)							C(G)
583(N)	A			G		G							G
584(N)	A			G(G)		G(G)							G(G)
615(Y)	C			T		T							T
630(S)	C			T									
633(Q)	G			A		A						A	A
636(E)	A			G									G
640(K)	A			A(K)									
642(K)	A			G(K)		G							G
644(C)	G										C(S)		
660(N)	T			C									
663(N)	T					C							C
669(L)	G			A									A

**699(V)**	**T**			A		A							A
**705(L)**	**G**			A		A							A
**706(P)**	**C**			T(S)		T(S)							T(S)
**708(P)**	**A**			G(S)		(S)							(S)
**723(S)**	**G**			T		T							T
**729(Q)**	**G**			A									
**734(K)**	**A**					C(T)							C(T)
**761(I)**	**T**												C(T)
**763(V)**	**G**												A(I)
**771(K)**	**A**												G
**787(E)**	**G**									A(K)			

[1] Nt = nucleotide; [2] Accession numbers from National Center for Biotechnological Information (NCBI)
